# (*Z*)-4-Benzyl­idene-3-methyl­isoxazol-5(4*H*)-one

**DOI:** 10.1107/S1600536812041311

**Published:** 2012-10-10

**Authors:** Chandra, N. Srikantamurthy, S. Jeyaseelan, K. B. Umesha, K. Palani, M. Mahendra

**Affiliations:** a Department of Studies in Physics, Manasagangotri, University of Mysore, Mysore 570 006, India; b Department of Chemistry, Yuvaraja’s College, University of Mysore, Mysore 570 005, India; c Department of Physics, St Philomena’s College, Mysore, India; d SER-CAT, APS, Argonne National Laboratory, Argonne, IL-60439, USA

## Abstract

In the title compound C_11_H_9_NO_2_, the phenyl and isoxazole rings are almost coplanar, making a dihedral angle of 1.14 (9)°. This planarity is also assisted by an intra­molecular C—H⋯O hydrogen bond between the phenyl ring and the carbonyl O atom. In the crystal, weak C—H⋯O inter­actions generate a layered structure parallel to the *ac* plane.

## Related literature

For the biological and therapeutic importance of isoxazoles, see: Kang *et al.* (2000 ▶); Conti *et al.* (1998 ▶); Changtam *et al.* (2010 ▶); Kwon *et al.*, (1995 ▶); Abbiati *et al.* (2003 ▶). For bond-length and angle data in a related structure, see: Wolf *et al.* (1995 ▶).
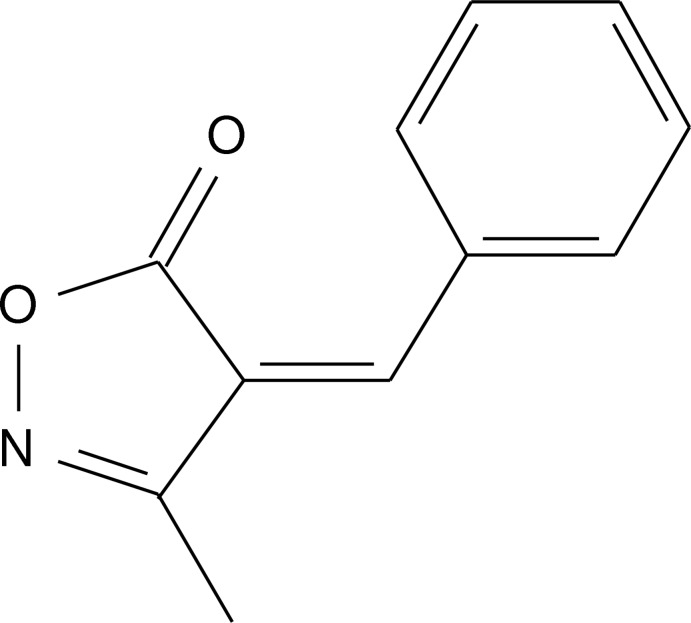


## Experimental

### 

#### Crystal data

C_11_H_9_NO_2_
*M* *_r_* = 187.19
Monoclinic, 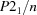

*a* = 12.144 (4) Å
*b* = 6.734 (2) Å
*c* = 12.333 (4) Å
β = 114.589 (5)°
*V* = 917.1 (5) Å^3^
*Z* = 4
Mo *K*α radiation
μ = 0.10 mm^−1^
*T* = 293 K
0.30 × 0.25 × 0.20 mm

#### Data collection

Bruker APEXII CCD area-detector diffractometer
6722 measured reflections
1610 independent reflections
1352 reflections with *I* > 2σ(*I*)
*R*_int_ = 0.016

#### Refinement

*R*[*F* ^2^ > 2σ(*F* ^2^)] = 0.042
*wR*(*F* ^2^) = 0.111
*S* = 1.07
1610 reflections
128 parameters
H-atom parameters constrained
Δρ_max_ = 0.19 e Å^−3^
Δρ_min_ = −0.14 e Å^−3^

### 

Data collection: *APEX2* (Bruker, 2009 ▶); cell refinement: *SAINT* (Bruker, 2009 ▶); data reduction: *SAINT*; program(s) used to solve structure: *SHELXS97* (Sheldrick, 2008 ▶); program(s) used to refine structure: *SHELXL97* (Sheldrick, 2008 ▶); molecular graphics: *PLATON* (Spek, 2009 ▶); software used to prepare material for publication: *SHELXL97*.

## Supplementary Material

Crystal structure: contains datablock(s) global, I. DOI: 10.1107/S1600536812041311/sj5268sup1.cif

Structure factors: contains datablock(s) I. DOI: 10.1107/S1600536812041311/sj5268Isup2.hkl

Supplementary material file. DOI: 10.1107/S1600536812041311/sj5268Isup3.cml

Additional supplementary materials:  crystallographic information; 3D view; checkCIF report

## Figures and Tables

**Table 1:** Hydrogen-bond geometry (Å, °)

*D*—H⋯*A*	*D*—H	H⋯*A*	*D*⋯*A*	*D*—H⋯*A*
C10—H10⋯O1	0.93	2.21	3.042 (2)	149
C7—H7*C*⋯O6^i^	0.96	2.61	3.297 (2)	129
C8—H8⋯O1^i^	0.93	2.72	3.574 (2)	154
C14—H14⋯O1^i^	0.93	2.68	3.526 (2)	151

Symmetry code: (i) 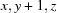.

**Fig. 1.**
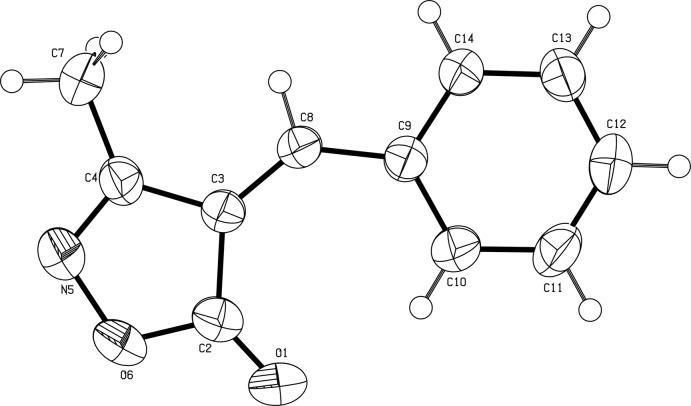
Perspective diagram of the molecule with 50% probability displacement ellipsoids.

**Fig. 2.**
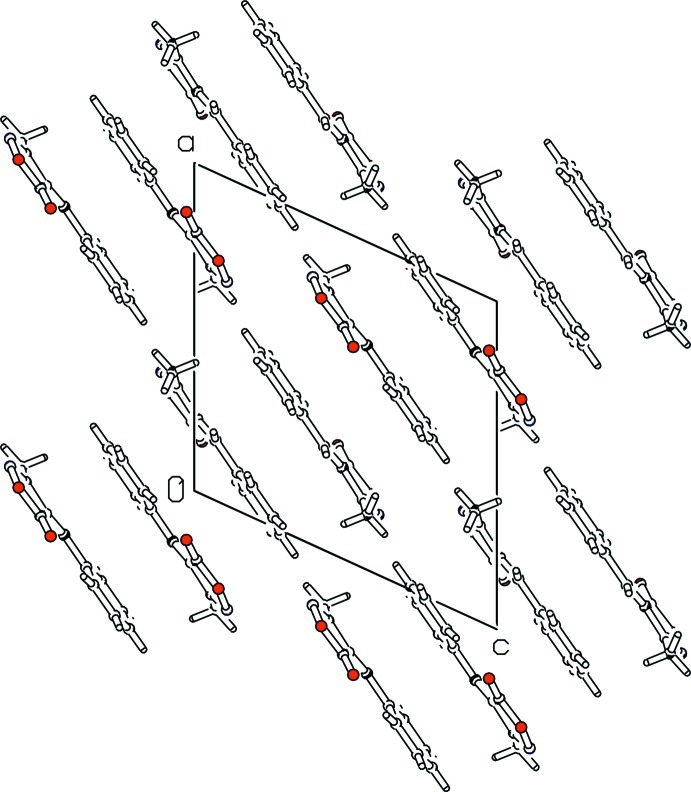
Packing diagram of the molecule viewed down the b axis.

## supplementary crystallographic information

### Comment

Isoxazole and its derivatives represent one of the important classes of heterocyclic compounds. These derivatives are employed in the area of pharmaceuticals and demonstrate therapeutic properties such as anti-tumor (Kang *et al.*, 2000), hypoglycemic (Conti *et al.*, 1998), anti-mycobacterial (Changtam *et al.*, 2010) and anti-inflammatory activity (Kwon *et al.*, 1995). In addition, isoxazole derivatives serve as versatile building blocks in organic synthesis (Abbiati *et al.*, 2003). With this extensive background of isoxazole derivatives, we have synthesized the title compound to study its crystal structure.

In the molecular structure of the title compound (Fig. 1), the dihedral angle between the phenyl ring (C9/C10/C11/C12/C13/C14) and isoxazole ring (C1/C3/C4/N5/O6) is 1.14 (9)°. The isoxazole moiety is in a *syn-periplanar* conformation with respect to the phenyl ring, as indicated by the torsion angle value of 0.5 (2)°. The bond lengths and angles agree with those reported for a related structure (Wolf *et al.*, 1995). There are no classic hydrogen bonds. In the crystal structure weak C—H···O hydrogen bonds link molecules into sheets Table 1. The packing diagram viewed down the *b* axis shows a layered stacking feature (Fig. 2).

### Experimental

A mixture of benzaldehyde oxime (1 mmol), ethyl acetoacetate (2 mmol) and anhydrous zinc chloride (0.1 mmol) were taken in a 10 ml round bottomed flask and contents were gradually heated to 120°C without any solvent for about one hour. After completion of the reaction (as indicated by TLC), the mixture was cooled to room temperature and methanol was added with stirring for about 30 min; the solids thus obtained were filtered and recrystallized from hot ethanol.

### Refinement

H atoms were placed at idealized positions and allowed to ride on their parent atoms with C–H distances in the range of 0.93 to 0.96 Å; *U*_iso_(H) = 1.2-1.5*U*_eq_(carrier atom) for all H atoms.

### Crystal data

**Table:**

C_11_H_9_NO_2_	*F*(000) = 392
*M**_r_* = 187.19	*D*_x_ = 1.356 Mg m^−^^3^
Monoclinic, *P*2_1_/*n*	Mo *K*α radiation, λ = 0.71073 Å
Hall symbol: -P 2yn	Cell parameters from 1610 reflections
*a* = 12.144 (4) Å	θ = 2.0–25.0°
*b* = 6.734 (2) Å	µ = 0.10 mm^−^^1^
*c* = 12.333 (4) Å	*T* = 293 K
β = 114.589 (5)°	Block, yellow
*V* = 917.1 (5) Å^3^	0.30 × 0.25 × 0.20 mm
*Z* = 4	

### Data collection

**Table:**

Bruker APEXII CCD area-detector diffractometer	*R*_int_ = 0.016
ω and φ scans	θ_max_ = 25.0°, θ_min_ = 2.0°
6722 measured reflections	*h* = −14→14
1610 independent reflections	*k* = −7→7
1352 reflections with *I* > 2σ(*I*)	*l* = −14→14

### Refinement

**Table:**

Refinement on *F*^2^	Primary atom site location: structure-invariant direct methods
Least-squares matrix: full	Secondary atom site location: difference Fourier map
*R*[*F*^2^ > 2σ(*F*^2^)] = 0.042	Hydrogen site location: inferred from neighbouring sites
*wR*(*F*^2^) = 0.111	H-atom parameters constrained
*S* = 1.07	*w* = 1/[σ^2^(*F*_o_^2^) + (0.0548*P*)^2^ + 0.1944*P*] where *P* = (*F*_o_^2^ + 2*F*_c_^2^)/3
1610 reflections	(Δ/σ)_max_ < 0.001
128 parameters	Δρ_max_ = 0.19 e Å^−^^3^
0 restraints	Δρ_min_ = −0.14 e Å^−^^3^

### Special details

**Table:**

Geometry. Bond distances, angles *etc*. have been calculated using the rounded fractional coordinates. All su's are estimated from the variances of the (full) variance-covariance matrix. The cell e.s.d.'s are taken into account in the estimation of distances, angles and torsion angles
Refinement. Refinement on *F*^2^ for ALL reflections except those flagged by the user for potential systematic errors. Weighted *R*-factors *wR* and all goodnesses of fit *S* are based on *F*^2^, conventional *R*-factors *R* are based on *F*, with *F* set to zero for negative *F*^2^. The observed criterion of *F*^2^ > σ(*F*^2^) is used only for calculating -*R*-factor-obs *etc*. and is not relevant to the choice of reflections for refinement. *R*-factors based on *F*^2^ are statistically about twice as large as those based on *F*, and *R*-factors based on ALL data will be even larger.

### Fractional atomic coordinates and isotropic or equivalent isotropic displacement parameters (Å^2^)

**Table:**

	*x*	*y*	*z*	*U*_iso_*/*U*_eq_	
O1	0.33893 (13)	0.04132 (18)	0.47425 (13)	0.0680 (5)	
O6	0.23528 (13)	0.11975 (18)	0.58010 (12)	0.0703 (5)	
N5	0.18345 (15)	0.2949 (2)	0.60829 (15)	0.0632 (6)	
C2	0.29158 (16)	0.1691 (2)	0.50821 (15)	0.0505 (6)	
C3	0.27739 (13)	0.3852 (2)	0.48985 (13)	0.0387 (5)	
C4	0.20890 (14)	0.4425 (2)	0.55692 (14)	0.0450 (5)	
C7	0.16786 (17)	0.6440 (3)	0.57095 (17)	0.0594 (7)	
C8	0.31588 (13)	0.5159 (2)	0.42957 (13)	0.0392 (5)	
C9	0.38358 (13)	0.4957 (2)	0.35687 (13)	0.0391 (5)	
C10	0.42882 (16)	0.3176 (2)	0.33333 (15)	0.0514 (6)	
C11	0.49210 (17)	0.3185 (3)	0.26289 (17)	0.0593 (7)	
C12	0.51257 (17)	0.4919 (3)	0.21543 (16)	0.0556 (6)	
C13	0.47001 (15)	0.6689 (3)	0.23875 (15)	0.0522 (6)	
C14	0.40617 (14)	0.6711 (2)	0.30883 (14)	0.0452 (5)	
H7A	0.13330	0.64020	0.62820	0.0890*	
H7B	0.10800	0.68910	0.49560	0.0890*	
H7C	0.23560	0.73320	0.59810	0.0890*	
H8	0.29440	0.64640	0.43610	0.0470*	
H10	0.41620	0.19900	0.36510	0.0620*	
H11	0.52150	0.19940	0.24720	0.0710*	
H12	0.55500	0.48960	0.16770	0.0670*	
H13	0.48420	0.78670	0.20740	0.0630*	
H14	0.37770	0.79120	0.32430	0.0540*	

### Atomic displacement parameters (Å^2^)

**Table:**

	*U*^11^	*U*^22^	*U*^33^	*U*^12^	*U*^13^	*U*^23^
O1	0.0942 (10)	0.0354 (7)	0.0893 (10)	0.0065 (6)	0.0529 (8)	0.0014 (6)
O6	0.1040 (11)	0.0394 (7)	0.0916 (10)	−0.0073 (7)	0.0646 (9)	0.0076 (6)
N5	0.0818 (11)	0.0514 (9)	0.0777 (11)	−0.0065 (8)	0.0544 (9)	0.0020 (8)
C2	0.0602 (10)	0.0383 (9)	0.0576 (10)	−0.0049 (8)	0.0291 (9)	0.0003 (7)
C3	0.0401 (8)	0.0349 (8)	0.0435 (8)	−0.0026 (6)	0.0197 (7)	−0.0019 (6)
C4	0.0466 (9)	0.0452 (9)	0.0490 (9)	−0.0065 (7)	0.0258 (8)	−0.0008 (7)
C7	0.0701 (12)	0.0539 (11)	0.0733 (12)	0.0062 (9)	0.0488 (10)	−0.0012 (9)
C8	0.0415 (8)	0.0343 (8)	0.0443 (8)	0.0008 (6)	0.0203 (7)	−0.0021 (6)
C9	0.0387 (8)	0.0401 (8)	0.0400 (8)	−0.0011 (6)	0.0178 (7)	−0.0026 (6)
C10	0.0615 (11)	0.0394 (9)	0.0615 (10)	−0.0027 (8)	0.0339 (9)	−0.0073 (7)
C11	0.0681 (12)	0.0532 (11)	0.0706 (12)	0.0000 (9)	0.0427 (10)	−0.0172 (9)
C12	0.0555 (10)	0.0707 (12)	0.0503 (10)	−0.0019 (9)	0.0317 (8)	−0.0070 (8)
C13	0.0547 (10)	0.0566 (11)	0.0531 (10)	0.0014 (8)	0.0303 (8)	0.0088 (8)
C14	0.0480 (9)	0.0438 (9)	0.0498 (9)	0.0057 (7)	0.0263 (8)	0.0036 (7)

### Geometric parameters (Å, º)

**Table:**

O1—C2	1.203 (2)	C11—C12	1.374 (3)
O6—N5	1.446 (2)	C12—C13	1.376 (3)
O6—C2	1.367 (3)	C13—C14	1.381 (3)
N5—C4	1.284 (2)	C7—H7A	0.9600
C2—C3	1.472 (2)	C7—H7B	0.9600
C3—C4	1.448 (2)	C7—H7C	0.9600
C3—C8	1.355 (2)	C8—H8	0.9300
C4—C7	1.480 (3)	C10—H10	0.9300
C8—C9	1.453 (2)	C11—H11	0.9300
C9—C10	1.399 (2)	C12—H12	0.9300
C9—C14	1.399 (2)	C13—H13	0.9300
C10—C11	1.379 (3)	C14—H14	0.9300
			
O1···C10	3.042 (2)	C13···C2^vi^	3.364 (3)
O6···C7^i^	3.297 (3)	C13···C3^vi^	3.471 (3)
O1···H10	2.2100	C14···C2^iii^	3.582 (3)
O1···H14^i^	2.6800	C2···H10	2.7700
O1···H8^i^	2.7200	C3···H10	2.9900
O6···H7C^i^	2.6100	C7···H8	2.6900
O6···H12^ii^	2.9100	C8···H7C	3.0200
N5···H12^ii^	2.7600	C11···H7B^iv^	3.0300
C2···C10	3.380 (3)	C11···H7C^iii^	3.0500
C2···C14^iii^	3.582 (3)	H7A···H13^vii^	2.4400
C2···C13^iv^	3.364 (3)	H7B···C11^vi^	3.0300
C2···C13^iii^	3.434 (3)	H7C···O6^v^	2.6100
C3···C13^iii^	3.493 (3)	H7C···C8	3.0200
C3···C13^iv^	3.471 (3)	H7C···H8	2.4600
C3···C12^iii^	3.562 (3)	H7C···C11^iii^	3.0500
C4···C12^iii^	3.408 (3)	H8···O1^v^	2.7200
C7···O6^v^	3.297 (3)	H8···C7	2.6900
C8···C10^iii^	3.446 (3)	H8···H7C	2.4600
C8···C9^iii^	3.502 (3)	H8···H14	2.2400
C9···C9^iii^	3.486 (2)	H10···O1	2.2100
C9···C8^iii^	3.502 (3)	H10···C2	2.7700
C10···C8^iii^	3.446 (3)	H10···C3	2.9900
C10···C2	3.380 (3)	H12···O6^viii^	2.9100
C10···O1	3.042 (2)	H12···N5^viii^	2.7600
C12···C4^iii^	3.408 (3)	H13···H7A^ix^	2.4400
C12···C3^iii^	3.562 (3)	H14···O1^v^	2.6800
C13···C3^iii^	3.493 (3)	H14···H8	2.2400
C13···C2^iii^	3.434 (3)		
			
N5—O6—C2	110.06 (12)	C9—C14—C13	121.09 (15)
O6—N5—C4	107.14 (16)	C4—C7—H7A	109.00
O1—C2—O6	119.51 (14)	C4—C7—H7B	109.00
O1—C2—C3	134.11 (18)	C4—C7—H7C	109.00
O6—C2—C3	106.38 (14)	H7A—C7—H7B	109.00
C2—C3—C4	103.52 (13)	H7A—C7—H7C	109.00
C2—C3—C8	132.98 (16)	H7B—C7—H7C	109.00
C4—C3—C8	123.48 (13)	C3—C8—H8	113.00
N5—C4—C3	112.89 (14)	C9—C8—H8	113.00
N5—C4—C7	119.35 (17)	C9—C10—H10	120.00
C3—C4—C7	127.76 (15)	C11—C10—H10	120.00
C3—C8—C9	133.69 (13)	C10—C11—H11	119.00
C8—C9—C10	125.46 (14)	C12—C11—H11	119.00
C8—C9—C14	116.32 (13)	C11—C12—H12	120.00
C10—C9—C14	118.22 (15)	C13—C12—H12	120.00
C9—C10—C11	119.80 (15)	C12—C13—H13	120.00
C10—C11—C12	121.27 (18)	C14—C13—H13	120.00
C11—C12—C13	119.77 (19)	C9—C14—H14	119.00
C12—C13—C14	119.85 (17)	C13—C14—H14	119.00
			
C2—O6—N5—C4	−0.8 (2)	C8—C3—C4—C7	0.9 (3)
N5—O6—C2—O1	−179.11 (17)	C2—C3—C4—N5	−0.07 (19)
N5—O6—C2—C3	0.72 (19)	C3—C8—C9—C10	−1.0 (3)
O6—N5—C4—C3	0.5 (2)	C3—C8—C9—C14	−179.86 (17)
O6—N5—C4—C7	−179.50 (15)	C8—C9—C10—C11	−179.85 (17)
O6—C2—C3—C4	−0.41 (18)	C14—C9—C10—C11	−1.0 (3)
O1—C2—C3—C4	179.4 (2)	C8—C9—C14—C13	179.80 (15)
O1—C2—C3—C8	−1.8 (4)	C10—C9—C14—C13	0.8 (2)
O6—C2—C3—C8	178.46 (17)	C9—C10—C11—C12	0.4 (3)
C2—C3—C4—C7	179.93 (17)	C10—C11—C12—C13	0.4 (3)
C8—C3—C4—N5	−179.08 (16)	C11—C12—C13—C14	−0.5 (3)
C2—C3—C8—C9	1.8 (3)	C12—C13—C14—C9	−0.1 (3)
C4—C3—C8—C9	−179.54 (16)		

Symmetry codes: (i) *x*, *y*−1, *z*; (ii) *x*−1/2, −*y*+1/2, *z*+1/2; (iii) −*x*+1, −*y*+1, −*z*+1; (iv) −*x*+1/2, *y*−1/2, −*z*+1/2; (v) *x*, *y*+1, *z*; (vi) −*x*+1/2, *y*+1/2, −*z*+1/2; (vii) *x*−1/2, −*y*+3/2, *z*+1/2; (viii) *x*+1/2, −*y*+1/2, *z*−1/2; (ix) *x*+1/2, −*y*+3/2, *z*−1/2.

### Hydrogen-bond geometry (Å, º)

**Table:**

*D*—H···*A*	*D*—H	H···*A*	*D*···*A*	*D*—H···*A*
C10—H10···O1	0.93	2.21	3.042 (2)	149
C7—H7*C*···O6^v^	0.96	2.61	3.297 (2)	129
C8—H8···O1^v^	0.93	2.72	3.574 (2)	154
C14—H14···O1^v^	0.93	2.68	3.526 (2)	151

Symmetry code: (v) *x*, *y*+1, *z*.
